# Supplementary material for: PM2.5-Bound Polycyclic Aromatic Hydrocarbons: Sources and Health Risk during Non-Heating and Heating Periods (Tangshan, China)
Source: Int J Environ Res Public Health. 2020 Jan 11;17(2):483. doi: 10.3390/ijerph17020483 (PMC7014208; doi:10.3390/ijerph17020483)
Supplement: Supplementary file 1 [file ijerph-17-00483-s001.pdf]

Table S1. Chemical structures and groups of eighteen PAHs

PAHs species	Abbreviation	Chemical structures	Molecular weight	Rings
Naphthalene	Nap	$C_{10}H_8$	128	2
Acenaphthylene	Acy	$C_{12}H_8$	152	3
Acenaphthene	Ace	$C_{12}H_{10}$	154	3
Fluorene	Flu	$C_{13}H_{10}$	166	3
Phenanthrene	Phe	$C_{14}H_{10}$	178	3
Anthracene	Ant	$C_{14}H_{10}$	178	3
Fluoranthene	Fln	$C_{16}H_{10}$	202	4
Pyrene	Pyr	$C_{16}H_{10}$	202	4
Chrysene	Chr	$C_{18}H_{12}$	228	4
Benz[a]anthracene	BaA	$C_{18}H_{12}$	228	4
Benz[b]fluoranthene	BbF	$C_{20}H_{12}$	252	5
Benz[k]fluoranthene	BkF	$C_{20}H_{12}$	252	5
Benzo[j]fluoranthene	BjF	$C_{20}H_{12}$	252	5
Benzo[a]pyrene	BaP	$C_{20}H_{12}$	252	5
Benzo[e]pyrene	BeP	$C_{20}H_{12}$	252	5
Indeno[1,2,3-cd]pyrene	InD	$C_{22}H_{12}$	276	6
Benzo[g,h,i]-perylene	BghiP	$C_{22}H_{12}$	276	6
Dibenzo[a,h]anthracene	DbA	$C_{22}H_{14}$	278	6

Table S2. Mann-Whitney U test of $PM_{2.5}$, $\sum_{18}$PAHs and BaP in non-heating and heating periods

Variables	Non-heating period	Heating period	*p* value
$PM_{2.5}$	79 (115)	106 (175)	0.091
$\sum_{18}$PAHs	185 (126)	282 (193)	0.003
BaP	3.64 (6.20)	60.4 (31.5)	< 0.001
Temperature (°C)	4.95 (12.4)	-1.25 (3.55)	0.023
Relative humidity (%)	66 (29.5)	66 (35.5)	0.862
Wind speed (m/s)	7.5 (2.75)	6.95 (4.33)	0.659

$p < 0.05$ for statistical difference.

Table S3. Spearman correlation of $\sum_{18}$PAHs and meteorological factors in samping periods

Variables	Temperature	Relative humidity (%)	Wind speed	$\sum_{18}$PAHs
Temperature	1	-0.033	-0.015	-0.428**
Relative humidity (%)		1	-0.695**	0.255
wind speed			1	0.113
$\sum_{18}$PAHs				1

*** Correlation is significant at the 0.01 level (two-tailed)*

Table S4. Diagnostic ratios of $PM_{2.5}$-bound PAHs and their corresponding values in present study

Diagnostic ratio	Non-heating period	Heating period	Ratio range	Sources	References
Ant/(Ant+Phe)	0.28	0.43	< 0.1	Petroleum	[49]
			> 0.1	Combustion	
Flu/(Flu+Pyr)	0.55	0.59	< 0.4	Petroleum input	[49,50]
			0.4-0.5	Liquid fossil fuel combustion	
			> 0.5	Grass, wood and coal combustion	
BaA/(BaA+Chr)	0.58	0.57	> 0.35	Petroleum combustion	[6,49]
			0.2-0.35	Grass, wood and coal combustion	
			< 0.2	Petroleum	
InD/(InD+BghiP)	0.21	0.15	< 0.2	Petroleum sources	[6]
			0.2-0.5	Petroleum combustion	
			> 0.5	Grass, wood and coal combustion	

Table S5. Average BaPeq concentrations of $PM_{2.5}$-bound PAHs (ng/m^3)

PAHs species	Toxic equivalency factor (TEF)	BaPeq concentration (ng/m^3)	
		Non-heating period	Heating period
Nap	0.001	0.014	0.033
Acy	0.001	0.024	0.043
Ace	0.001	0.006	0.012
Flu	0.001	0.001	0.003
Phe	0.001	0.005	0.005
Ant	0.01	0.045	0.035
Fln	0.001	0.005	0.017
Pyr	0.001	0.004	0.012
Chr	0.01	0.021	0.076
BaA	0.1	0.316	0.985
BbF	0.1	0.683	2.667
BkF	0.1	2.548	1.655
BjF	0.1	1.286	3.383
BaP	1	18.900	61.560
BeP	0.01	0.227	0.069
DbA	1	0.810	0.375
BghiP	0.01	0.517	0.239
InD	0.1	1.797	0.205
Total		27.2	71.4
BaP/Total (%)		69.5	85.3
Carcinogenicity index		2.99×10^{-5}	7.85×10^{-5}
